# Investigation into the Mode of Phosphate Activation in the 4-Hydroxy-4-Methyl-2-Oxoglutarate/4-Carboxy-4-Hydroxy-2-Oxoadipate Aldolase from *Pseudomonas putida* F1

**DOI:** 10.1371/journal.pone.0164556

**Published:** 2016-10-14

**Authors:** Scott Mazurkewich, Stephen Y. K. Seah

**Affiliations:** Department of Molecular and Cellular Biology, University of Guelph, Guelph, Ontario, Canada; Wageningen Universiteit, NETHERLANDS

## Abstract

The 4-hydroxy-4-methyl-2-oxoglutarate (HMG)/4-carboxy-4-hydroxy-2-oxoadipate (CHA) aldolase is the last enzyme of both the gallate and protocatechuate 4,5-cleavage pathways which links aromatic catabolism to central cellular metabolism. The enzyme is a class II, divalent metal dependent, aldolase which is activated in the presence of inorganic phosphate (P_*i*_), increasing its turnover rate >10-fold. This phosphate activation is unique for a class II aldolase. The aldolase pyruvate methyl proton exchange rate, a probe of the general acid half reaction, was increased 300-fold in the presence of 1 mM P_*i*_ and the rate enhancement followed saturation kinetics giving rise to a *K*_M_ of 397 ± 30 μM. Docking studies revealed a potential P_*i*_ binding site close to, or overlapping with, the proposed general acid water site. Putative P_*i*_ binding residues were substituted by site-directed mutagenesis which resulted in reductions of P_*i*_ activation. Significantly, the active site residue Arg-123, known to be critical for the catalytic mechanism of the enzyme, was also implicated in supporting P_*i*_ mediated activation.

## Introduction

Strains of *Pseudomonas* and *Sphingomonas* are well known for their capability to degrade a wide range of aromatic compounds including organic pollutants and lignin metabolites. In these species many aromatic compounds, such as phenanthrene, fluorene, and lignin metabolites are degraded to protocatechuate, a common metabolite which is further catabolized through either a 2,3-, 3,4-, or 4,5-cleavage pathway [1–6]. The 4-hydroxy-4-methyl-2-oxoglutarate (HMG)/4-carboxy-4-hydroxy-2-oxoadipate (CHA) aldolase is the final enzyme in the 4,5-cleavage pathway and catalyzes an essential step that connects the aromatic degradation pathway to central cellular metabolism [7]. The enzyme is a class II, divalent metal ion dependent, pyruvate aldolase which catalyzes the aldol cleavage of HMG and CHA into two molecules of pyruvate in the former substrate and a molecule each of pyruvate and oxaloacetate (OAA) in the later substrate. The HMG/CHA aldolase from *P*. *straminea* (formerly *Pseudomonas ochraceae*) is activated in the presence of inorganic phosphate (P_*i*_) increasing its relatively high turnover rate of 5.1 s^-1^ for CHA and 6.5 s^-1^ for HMG by 13- and 65-fold, respectively [8]. The DDG aldolase, a class II pyruvate aldolase unrelated to the HMG/CHA aldolase, was originally proposed to utilize P_*i*_ in its reaction mechanism based on the presence of P_*i*_ in the crystal structure of the enzyme [9]. However, kinetic analysis of a DDG aldolase homolog, HpaI, demonstrated that P_*i*_ is not required for maximal activity [10–13]. Therefore, to date, HMG/CHA aldolase is the only class II pyruvate aldolase that is activated by P_*i*_.

The presence of P_*i*_ shifted the optimum pH to more alkaline, but no gross structural changes of the *P*. *straminea* HMG/CHA aldolase were detected through circular dichroism [8]. Several enzymes such as malate dehydrogenase, heterodisulfide reductase, and dihydrodiol dehydrogenase, are also known to be activated in the presence of P_*i*_ [14–16]. However, the activation by P_*i*_ in these enzymes is minimal (< 3-fold) and may be attributable to changes in the ionic character of the solution. Defining how P_*i*_ interacts with and mediates the activation of the HMG/CHA aldolase has previously been impeded by a lack of structural information.

**Fig 1:**
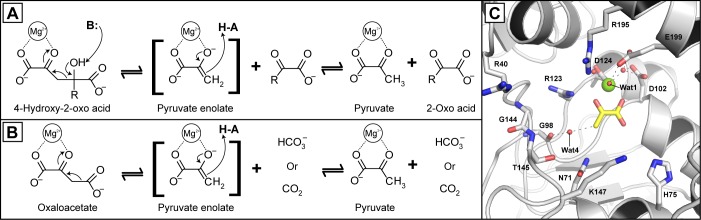
The HMG/CHA aldolase reactions and active site. (A) Aldol cleavage catalyzed by the HMG/CHA aldolase where for HMG the R group is -CH_3_ which yields 2 moles of pyruvate and for CHA the R group is -CH_2_COO^-^ yielding 1 mole each of pyruvate and oxaloacetate. (B) Oxaloacetate decarboxylase reaction catalyzed by the aldolase producing either carbon dioxide or bicarbonate. (C) Active site of the aldolase showing the pyruvate carbon in yellow sticks, the bound magnesium ion as a green sphere, and key water molecules as red spheres. The structural representation was generated in Pymol (version 1.7) using atomic coordinates of the HMG/CHA aldolase (PDB ID: 3NOJ).

The first structure of an HMG/CHA aldolase was recently solved. The enzyme from *Pseudomonas putida* F1 contains an αββα sandwich fold which is structurally distinct from the previously identified class II pyruvate aldolases of 4-hydroxy-2-oxoheptane-1,7-dioate aldolase (HpcH or HpaI), 4-hydroxy-2-oxopentanoate aldolase (DmpG or BphI), and 2-dehydro-3-deoxy-galactarate aldolase [9,17–19]. The mechanism of the HMG/CHA aldolase, and other class II pyruvate aldolases, is proposed to proceed through a two-step general acid/base mechanism with a pyruvate enolate intermediate (Fig 1A) [9,12,19,20]. The HMG/CHA aldolase, and other class II pyruvate aldolases, possesses oxaloacetate (OAA) decarboxylase activity owing to a common pyruvate enolate intermediate in both the aldolase and decarboxylase reactions (Fig 1B). Contrary to the other conserved class II pyruvate aldolases, the active site of the HMG/CHA aldolase is quite large relative to its substrate and water molecules are proposed as the acid and base in the enzyme’s catalytic mechanism (Fig 1C) [19].

The αββα sandwich fold of the HMG/CHA aldolase is structurally similar to the regulator of RNase E activity A (RraA) from *Escherichia coli*, a protein which binds to and modulates the *E*. *coli* RNA degradosome [19]. Gene copies of an HMG/CHA aldolase or RraA are found across all kingdoms of life, even in those who lack canonical bacterial aromatic degradation genes or RNase E. Most of the homologs contain one of two motifs required for metal binding and catalysis observed in the aldolase and thus likely function as class II pyruvate aldolases [21]. The HMG/CHA aldolase is also structurally related to the phospho-histidine (PH) domain found in both enzyme I of the phosphotransferase system (EI:PTS) and pyruvate phosphate dikinase (PPDK) [22,23]. Both these phosphotransferase (PT) proteins transfer the phosphoryl group from phosphoenolpyruvate to either a nucleotide in the case of PPDK or to a carrier protein in the case of EI:PTS, initiating a P_*i*_ transfer cascade leading to phosphorylation of carbohydrates and enabling cellular retention of the sugar in bacteria [24]. The conserved structural fold between the PH domain and the HMG/CHA aldolase may suggest a conserved P_*i*_ binding site amongst the proteins that has been previously unexplored.

Herein we report on the characterization of P_*i*_ activation in the HMG/CHA aldolase from *Pseudomonas putida* F1. Like the *P*. *straminea* enzyme, the *P*. *putida* F1 HMG/CHA aldolase is uncompetitively activated in the presence P_*i*_. Activation of the enzyme’s pyruvate methyl proton exchange rate in the presence of P_*i*_ is consistent with the polyatomic ion activating the general acid half reaction in aldolase mechanism. P_*i*_ was modelled in the active site of the HMG/CHA aldolase leading to proposed roles for key residues in the enzymes active. Results from mutagenesis and differential kinetic assays were consistent with the proposed model and support P_*i*_ mediated activation through the general acid mechanism.

## Experimental Procedures

### Chemicals and Enzymes

Racemic HMG and CHA were synthesized and purified as described previously [21]. L-lactate dehydrogenase (LDH, rabbit muscle), L-malate dehydrogenase (MDH, porcine heart), and oxaloacetic acid were from Sigma-Aldrich (Oakville, ON). Pfu polymerase was from Invitrogen (Burlington, ON). All other chemicals were analytical grade and were obtained from either Sigma-Aldrich or Fisher Scientific unless otherwise stated.

### Molecular Biology

The HMG/CHA aldolase gene from the *P*. *putida* F1 was previously inserted into pT7-7 [19]. Gene mutations for HMG/CHA aldolase variants were created using the QuikChange method [25]. Primers utilized for mutagenesis are listed in S1 Table. All modified plasmids were transformed into *E*. *coli* DH5α for propagation and the gene mutations were confirmed by DNA sequencing at the Guelph Molecular Supercenter (University of Guelph). The expression and purification of the HMG/CHA aldolase, including the variants, were as previously described [19]. Enzymes were treated with chelex to remove exogenous and complexed metals with the apo-enzymes reconstituted with the 250 μM MgCl_2_ for at least 30 minutes prior to enzyme assays [21]. Protein concentrations were determined by the Bradford assay using bovine serum albumin as standards [26]. SDS-PAGE was performed and stained with Coomassie Blue to assess protein purity according to established procedures [27].

### Enzyme Assays

All kinetics assays were performed at least in duplicate at 25°C using a Varian Cary 3 spectrophotometer equipped with a thermostatted cuvette holder. The HMG aldol cleavage and OAA decarboxylase activities were observed by coupling pyruvate formation to NADH oxidation using L-lactate dehydrogenase (LDH). Similarly, the CHA aldol cleavage activity was monitored by coupling oxaloacetate formation to NADH oxidation using L-malate dehydrogenase. NADH oxidation was monitored at 340 nm, and the extinction coefficient of NADH was taken to be 6220 M^-1^cm^-1^. Assays of 1 mL were completed in 0.1 M HEPES pH 8.0 supplemented with 1 mM MgCl_2_. Reactions were monitored for at least 2 minutes in the absence of enzyme for determination of the background rate of degradation. Enzyme was added in sufficient quantities to ensure at least a 2-fold change in the rate of substrate turnover from the background rate. Enzyme velocities were determined by subtraction of the rate background degradation from the rate observed in the enzyme catalyzed reaction. Stock solutions of Na_3_PO_4_ Na_3_AsO_4_, Na_3_VO_4_, Na_2_SO_4_, or Na_2_MoO_4_ were prepared with buffer at the assay pH. All kinetic data were fitted using non-linear regression in GraphPad Prism.

### Pyruvate Methyl Proton Exchange Assays

Assays for the determination of the HMG/CHA aldolase catalyzed pyruvate methyl proton exchange rate were determined similarly to those completed previously [19]. Briefly, assays of 600 μL were completed in D_2_O with 20 mM MOPS buffer at a pD of 8.0 (where pD = pH + 0.4 and the pH was measured to 8.0) with 30 mM sodium pyruvate, 1 mM MgCl_2_. Standard assays for P_*i*_ activation were completed with 1 mM sodium phosphate, at pD 8.0, and the rate enhancement work was completed with increasing P_*i*_ concentrations. All reagents were anhydrous and resuspended in 98% D_2_O from Cambridge Isotopes Laboratories Inc. (Andover, MA). Reactions were initiated with the addition of enzyme which had been prepared in 20 mM MOPS buffer pD of 8.0 with 1 mM MgCl_2_. The rate of pyruvate methyl proton exchange was detected with ^1^H NMR, in a Bruker Avance 600 MHz spectrometer at 25°C using a 5.0 mm NMR tube, with measurements taken every 2 minutes for 20 minutes. The rate of decrease in the pyruvate methyl proton signal (2.38 ppm) was determined by comparison to the MOPS methylene proton signal (2.05 ppm) which did not change during the course of the assay. The rate of decrease in the pyruvate methyl proton signal was fit by least squares regression of the natural log of the decrease in signal over time. The resulting rate of decrease in the methyl proton signal due to enzyme was determined by subtraction of the rate of observed in a no enzyme control using Eq 1. The enzymatic methyl proton exchange rate was then determined using Eq 2 where the concentration of pyruvate and enzyme are in units of mM, giving *V*_exchange_ in units of s^-1^. For determination of the rate enhancement due to P_*i*_, assays were completed with increasing P_*i*_ concentrations with the rate of the enzyme catalyzed exchange in the absence of P_*i*_ taken as zero and exchange rates determined in the presence of P_*i*_ fit to the Michaelis-Menten equation in GraphPad Prism 5.

$$k_{obs}=k_{enzyme}-k_{control}$$ (1)

$$V_{exchange}=\frac{3k_{obs}\left[\text{pyruvate}\right]}{\left[\text{enzyme}\right]}$$ (2)

### Modelling of P_i_ in the HMG/CHA Aldolase

Docking of phosphoric acid ligand was completed with Rosetta Online (ROSIE) [28,29]. The atomic coordinates of the solvent-free, holo-HMG/CHA aldolase (PDB ID: 3NOJ) was used as the template and the bound magnesium ion was given a 2^+^ formal charge. A 7 Å search radius from the center of the pocket (coordinates X = 11.7, Y = 21.0, and Z = 57.4) was utilized and over 1500 structures were generated. All other search parameters were set to default conditions. The top 100 poses containing the lowest interface delta score were chosen for clustering and further analyses [28,30]. Structure figures were prepared using PyMol v1.4.1 (Schrödinger, LLC).

## Results

### HMG/CHA Aldolase Activation by P_i_

The effect of P_*i*_ on the steady state kinetic parameters for the *P*. *putida* F1 HMG/CHA aldolase catalyzed reactions was assessed. A ~10-fold increase in both the *k*_cat_ and *K*_M_ is observed in the retro aldol reaction with CHA and HMG (Table 1). Only a ~2-fold increase in both *k*_cat_ and *K*_M_ is observed in the OAA decarboxylase reaction in the presence of P_*i*_. The results with HMG and OAA are similar to those of the *P*. *straminea* HMG/CHA aldolase previously reported [8]. However, only a ~10-fold activation is observed with CHA in the *P*. *putida* F1 enzyme opposed to the 55-fold activation observed with the *P*. *straminea* enzyme. Similar to the *P*. *straminea* enzyme, the *P*. *putida* F1 displays uncompetitive activation with P_*i*_ whose presence leads to increases in both *k*_cat_ and *K*_M_ values for the catalyzed reactions. Also similar to the *P*. *straminea* enzyme, assays completed with 2 mM Na_3_AsO_4_ and Na_3_VO_4_ gave the same degree of activation as that of P_*i*_. Neither 2 mM Na_2_SO_4_ nor Na_2_MoO_4_ activated the enzyme. The activity of the enzyme in the presence Na_2_SO_4_ up to 100 mM did not decrease the rate enhancement by P_*i*_ (2 mM) indicating the enzyme’s specificity for P_*i*_.

**Table 1:** P_*i*_ activation with different substrates.

Substrate	Without P_*i*_				+ 2 mM P_*i*_			
	*K*_*m*_ (μM)	*K*_si_ (mM)	*k*_cat_ (s^-1^)	*k*_cat_/*K*_*m*_ (M^-1^s^-1^)	*K*_*m*_ (μM)	*K*_si_ (mM)	*k*_cat_ (s^-1^)	*k*_cat_/*K*_*m*_ (M^-1^s^-1^)
**OAA**	**298 ± 30**	**0.360 ± 0.049**	**1.86 ± 0.18**	**(6.24 ± 0.87) x 10^3^**	**741 ± 25**	**ND **	**3.16 ± 0.036**	**(4.26 ± 0.15) x 10^3^**
**HMG**	**188 ± 11**	**1.80 ± 0.091**	**15.6 ± 0.54**	**(8.30 ± 0.56) x 10^4^**	**1360 ± 110**	**ND **	**134 ± 8.1**	**(9.85 ± 1.0) x 10^4^**
**CHA**	**15.0 ± 1.3**	**ND**	**13.4 ± 0.31**	**(8.93 ± 0.80) x 10^5^**	**104 ± 9.9**	**ND**	**145 ± 1.3**	**(1.40 ± 0.13) x 10^6^**

ND indicates data which were not detected under the conditions utilized.

### Effects of P_i_ on Proton Exchange

The aldolase second half reaction, the protonation of the pyruvate enolate, can be probed through analysis of the enzymes ability to exchange the pyruvate methyl protons with bulk solvent (Table 2). The exchange rate catalyzed by the aldolase is 2 s^-1^ which is slower than that of the aldolase reaction (~13.5 s^-1^ for CHA and ~15.5 s^-1^ for HMG) but is comparable to the OAA decarboxylase reaction (~2 s^-1^). Proton exchange assays in the presence of 1 mM P_*i*_ resulted in an increase of 300-fold in the pyruvate methyl proton exchange rate indicating a role for P_*i*_ to activate through the general acid half reaction. The fold increase in the proton exchange rate due to increasing P_*i*_ concentrations was saturatable yielding a *K*_M_ of 397 ± 30 μM with a maximal increase in activity of 402 ± 17-fold (S1 Fig).

**Table 2:** Pyruvate methyl proton exchange rate of the wild type and variants of the HMG/CHA aldolase in the absence and presence of P_*i*_.

Enzyme	Binding Site	- P_*i*_		+ 1mM P_*i*_	
		Exchange Rate (s^-1^)	Fold Change from Wt	Exchange Rate (s^-1^)	Fold activation
**Wt**	**-**	**2.02 ± 0.039**	**-**	**604 ± 8.2**	**298 ± 9.8**
**R40A**	**1**	**2.14 ± 0.078**	**0.946 ± 0.052**	**338 ± 11**	**158 ± 11**
**R123K**	**1**	**0.105 ± 0.024**	**-19.4 ± 4.8**	**1.24 ± 0.13**	**11.8 ± 4.1**
**G144V**	**1**	**2.54 ± 0.11**	**0.844 ± 0.051**	**19.6 ± 0.47**	**7.72 ± 0.84**
**T145A**	**1**	**1.39 ± 0.041**	**-1.45 ± 0.071**	**32.8 ± 1.3**	**23.6 ± 1.6**
**R195A**	**1**	**0.568 ± 0.026**	**-3.57 ± 0.23**	**31.1 ± 0.43**	**54.8 ± 3.3**
**N71A**	**1/2**	**0.325 ± 0.029**	**-6.23 ± 0.68**	**54.4 ± 1.2**	**167 ± 19**
**K147A**	**1/2**	**0.824 ± 0.053**	**-2.45 ± 0.21**	**33.6 ± 0.84**	**40.8 ± 3.7**
**H75A**	**2**	**1.98 ± 0.072**	**-0.980 ± 0.045**	**598 ± 12**	**302 ± 11**

### *In Silico* Docking Studies

**Fig 2:**
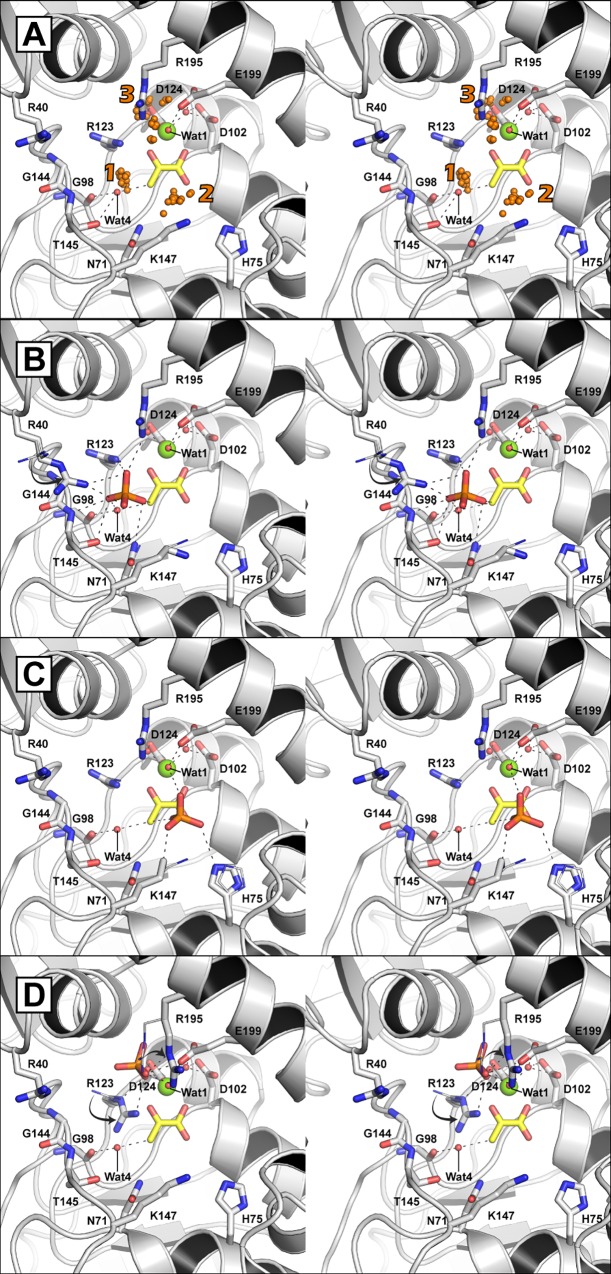
Docking of P_*i*_ into the active site of the HMG/CHA aldolase. The HMG/CHA aldolase as represented in Fig 1. (A) The center of mass of each P_*i*_ ion is shown as an orange sphere and the grouped putative binding sites are numbered. A representative binding mode for sites 1 (B), 2 (C), and 3 (D) are shown with the native structures residues as lines with significant positional changes indicated with arrows. Potential hydrogen bonds between P_*i*_ and the protein, defined by distances between 2.8 and 3.2 Å, are indicated by dashes.

Attempts to co-crystallize the HMG/CHA aldolase with P_*i*_ in conditions described previously were not successful and new crystallization conditions with the enzyme in the presence of P_*i*_ were not found [19]. Ligand docking studies with Rosetta were performed to ascertain potential P_*i*_ binding modes that could be evaluated by biochemical studies. The fully protonated phosphoric acid was used and 1500 poses were generated around a 7 Å radius from the center of the pyruvate binding pocket. Of these poses ~1000 contained interface delta scores less than zero and could be clustered into 3 groups based on the potential binding site (Fig 2A).

Site 1 is found close to, or overlapping, the proposed general acid water molecule. A representative binding mode from this cluster indicates potential interactions with Nη1 of Arg-123, Nδ of Asn71, backbone amide and the Oγ of Thr-145, the Nη1 of Arg-40, Nη1 of Arg-195, and both the proposed general acid and general base water molecules (Fig 2B). Site 2 is found ~4 Å angstroms from site 1 and is more central in the binding pocket. A representative pose indicates potential electrostatic interactions of P_*i*_ with Nε of His-75, Nζ of Lys-147, and the proposed general base metal ligated water (Fig 2C). Site 3 is found on the opposite side of the bound magnesium ion from the pyruvate molecule. Binding of P_*i*_ at this site is only accomplished by reorganization of the active site Arg-123 residue to a position out of line with the pyruvate carbonyl (Fig 2D). The positioning of the Arg-123 guanidinium is essential for catalysis through stabilization of the pyruvate enolate intermediate [19]. Further, binding of P_*i*_ at site 3 is also only accomplished through interactions of P_*i*_ with either one or both of the acidic metal binding residues Asp-124 and Glu-199. The protonation state at pH values where the enzyme is active, and activated, (pH values > 7) indicate that the P_*i*_ is likely in either the mono- or dianionic state and that charge repulsion with the acidic residues with the anionic P_*i*_ would result. Taken together, it is unlikely that P_*i*_ is capable of productively binding at site 3.

### Probing of Potential P_i_ Binding Sites

The residues comprising the putative P_*i*_ binding sites defined by docking studies were investigated through mutagenesis (Tables 2 and 3). The residues Asn-71 and Lys-147 are centrally located between the two proposed P_*i*_ binding sites 1 and 2. Enzyme variants of N71A and K147A had significant effects on the steady state kinetics of the aldolase HMG reaction with both variants decreasing the *k*_cat_ by 1114-fold indicating key functions for the residues but whose roles in the enzyme’s substrate binding and/or catalysis have not yet been elucidated. Both of the substitutions reduced the degree of P_*i*_ activation of the HMG cleavage reaction to less than 2-fold. The degree of P_*i*_ activation on the pyruvate methyl proton exchange rate was reduced relative to the wild type in the N71A and K147A variants by 2- and 7-fold, respectively.

**Table 3:** Steady state kinetic parameters of HMG lyase activity of variants of the HMG/CHA aldolase in the presence or absence of the P_*i*_.

Enzyme	Site	Without P_*i*_			+ 2 mM P_*i*_				
		*K*_*m*_ (μM)	*k*_cat_ (s^-1^)	*k*_cat_/*K*_*m*_ (M^-1^s^-1^)	*K*_*m*_ (μM)	*k*_cat_ (s^-1^)	*k*_cat_/*K*_*m*_ (M^-1^s^-1^)	Fold ↑ *k*_cat_	Fold ↑ *K*_*m*_
**Wt**	****	**188 ± 11**	**15.6 ± 0.54**	**(8.30 ± 0.49) x 10^4^**	**1360 ± 110**	**134 ± 8.1**	**(9.85 ± 1.0) x 10^4^**	**8.6**	**7.2**
**R40A**	**1**	**304 ± 27**	**6.70 ± 0.14**	**(2.20 ± 0.21) x 10^4^**	**3260 ± 340**	**36.6 ± 1.0**	**(1.12 ± 0.12) x 10^4^**	**5.5**	**11**
**R123K**	**1**	**3210 ± 210**	**0.0860 ± 0.0046**	**26.7 ± 2.3**	**3970 ± 25**	**0.0701 ± 0.0034**	**17.6 ± 0.86**	**0.82**	**1.2**
**G144V**	**1**	**1390 ± 160**	**0.215 ± 0.015**	**(1.55 ± 0.21) x 10^2^**	**6830 ± 440**	**0.394 ± 0.0090**	**57.7 ± 3.9**	**1.8**	**4.9**
**T145A**	**1**	**1080 ± 88**	**0.889 ± 0.057**	**(8.23 ± 0.85) x 10^2^**	**1780 ± 210**	**10.7 ± 0.33**	**(6.01 ± 0.73) x 10^3^**	**12**	**1.7**
**R195A**	**1**	**1370 ± 58**	**6.72 ± 0.19**	**(4.91 ± 0.25) x 10^3^**	**2890 ± 210**	**11.5 ± 0.67**	**(3.98 ± 0.37) x 10^3^**	**1.7**	**2.1**
**N71A**	**1/2**	**796 ± 70**	**0.0140 ± 0.0011**	**17.6 ± 2.1**	**1110 ± 82.7**	**0.0184 ± 0.00034**	**16.5 ± 1.3**	**1.3**	**1.4**
**K147A**	**1/2**	**2010 ± 210**	**0.0140 ± 0.00039**	**6.97 ± 0.75**	**3720 ± 340**	**0.0224 ± 0.00055**	**6.45 ± 0.61**	**1.6**	**1.8**
**H75A**	**2**	**389 ± 22**	**22.3 ± 0.75**	**(5.73 ± 0.38) x 10^4^**	**1210 ± 170**	**53.5 ± 1.7**	**(4.45 ± 0.64) x 10^4^**	**2.4**	**3.1**

Reactions were completed in the presence or absence of 2 mM sodium phosphate in 0.1 M HEPES buffer at pH 8.0.

Binding of P_*i*_ at the proposed site 2 requires His-75 to stabilize the polyatomic ion. The H75A variant had only a 2-fold increase in *K*_M_ and a 1.4-fold increase in *k*_cat_ for the HMG catalyzed reaction and did not have a significant effect on the pyruvate methyl proton exchange rate. The residue substitution resulted in only a 3.1-fold increase in *k*_cat_ in the presence of the P_*i*_, which is modest relative to the effects seen from substitution of residues from site 1. Furthermore, the H75A variant did not have an effect on the P_*i*_ activation of the pyruvate methyl proton exchange rate. Together, the results indicate that His-75 is not directly involved with P_*i*_ binding leading to enzyme activation and P_*i*_ binding at site 2 leading to enzyme activation is unlikely.

At site 1 two potential P_*i*_ binding modes are envisioned. In the first mode, P_*i*_ could bind in place of the proposed general acid water molecule, with the ion acting directly as the general acid. In the second mode, P_*i*_ could bind proximal to the proposed general acid water molecule and facilitate proton transfer between the water and the pyruvate enolate methylene. Thr-145 coordinates the proposed general acid water and substitution of the residue with alanine results in an 18-fold reduction in *k*_cat_ for the HMG catalyzed reaction relative to the wild type enzyme. The effect on the pyruvate methyl proton exchange rate, however, is minimal, only reducing the rate by 1.5-fold. The T145A variant is activated in the presence of P_*i*_ resulting in a 12-fold increase in *k*_cat_ for the HMG catalyzed reaction but only a 24-fold increase in the pyruvate methyl proton exchange rate. The lower enhancement of the pyruvate methyl proton exchange rate in the T145A variant relative to the wild type could suggest that P_*i*_ is ideally positioned by Thr-145 to act in lieu of the general acid water. However, this result is not conclusive and if P_*i*_ were proximally bound at site 1 the polyatomic ion could interact with Thr-145 and account for the decrease in activation by P_*i*_.

Along the same face of the pocket as the Thr-145, lies the Arg-40 residue which may be rotated towards the active site pocket to interact with P_*i*_ at site 1. The potential for the interaction by Arg-40 was assessed by both a R40A variant and a G144V variant; the glycine substitution to the larger valine side chain was thought to sterically restrict Arg-40 from interacting in the pocket. The R40A variant had only a 2-fold decrease in *k*_cat_ and was still activated to a similar extent as the wild type enzyme in the HMG aldolase cleavage reaction. The G144V variant, however, had a 10-fold increase in *K*_M_ and a 73-fold decrease in *k*_cat_ for the HMG cleavage reaction and the *k*_cat_ increased only 2-fold in the presence of P_*i*_. Neither of the R40A or G144V variants had a significant effect on the pyruvate methyl proton exchange rate, but the rate enhancement by P_*i*_ was reduced relative to the wild type by 2- and 39-fold, respectively. Together the results suggest the involvement of Arg-40 in P_*i*_ binding is minimal but inclusion of a substantial side chain at the Gly-144 position restricts activation by P_*i*_ likely by reducing the enzyme’s ability to bind the ion.

The guanidinium group of Arg-123 is proposed to stabilize the pyruvate enolate intermediate and the residues substitution by alanine reduces the enzyme’s activity below detection of the assay indicating the essentiality of this residue [19]. In accordance with this proposal, substitution of the Arg-123 with lysine, maintaining the electropositive character but modifying the chemical structure at this site, reduces the *k*_cat_ of the HMG catalyzed reaction by 181-fold and reduces the pyruvate methyl proton exchange rate by 20-fold. The proposed P_*i*_ binding at site 1 suggests that the Nη1 of Arg-123 may interact with the P_*i*_. There is no increase in *k*_cat_ for the HMG catalyzed reaction in the presence of P_*i*_ for the R123K variant and only a 10-fold increase in the pyruvate methyl proton exchange rate results in the presence of P_*i*_ with the variant. Together the results substantiate a role for Arg-123 in P_*i*_ binding leading to enzyme activation.

Arg-195 is close to the bound magnesium ion with its side chain projecting into the active site close, but at a 90° angle, to Arg-123 and may contribute to P_*i*_ binding in site 1. Compared to the wild type enzyme, the R195A substitution moderately effects the HMG cleavage reaction with a 7-fold increase in *K*_M_ and a 2-fold decrease in *k*_cat_. However, the activation of *k*_cat_ and the pyruvate methyl proton exchange rate by P_*i*_ is each reduced by 5-fold in the R195A variant relative to the wild type enzyme, consistent with the proposal of the residue contributing to P_*i*_ binding.

Taken together, the results of the mutagenesis and kinetic investigations support the proposal of P_*i*_ activating the general acid half reaction where the polyatomic ion is positioned by residues comprising site 1.

### Structural Comparison with Phosphotransferases

**Fig 3:**
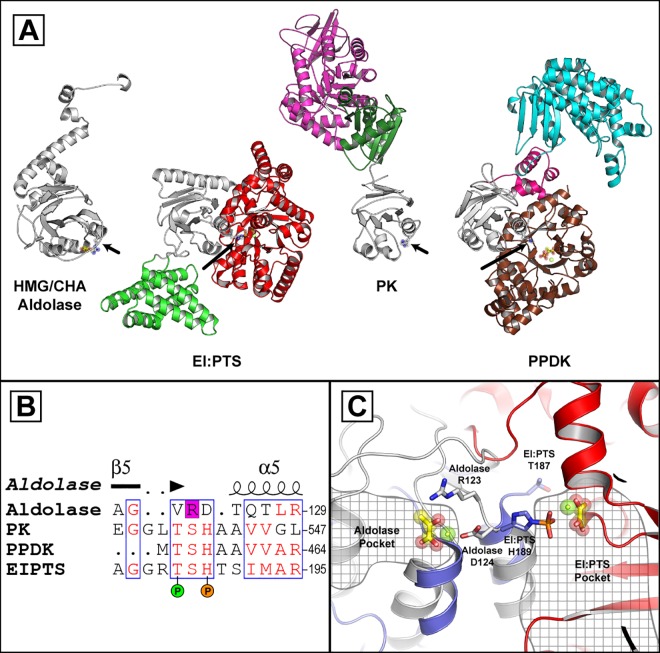
Comparison of the HMG/CHA aldolase with the PT proteins. (A) Overall structural comparison with the proteins oriented via structural alignment of the conserved domain which is coloured white with arrows pointing out the aldolase Arg-123 and transferase PH residue. The HMG/CHA aldolases Mg^2+^ and pyruvate are shown as a green sphere and yellow sticks, respectively. EI:PTS from *E*. *coli* (PDB: 2HWG) is shown with its PEP binding domain coloured red its and helical bundle, which supports HPr binding, coloured green. The EI:PTS Mg^2+^ and oxalate are shown as a green sphere and yellow sticks, respectively. PK from *Geobacillus strearothermophilus* (PDB: 2E28) is shown with its kinase domain coloured magenta its and effector domain coloured dark green. The PPDK from Maize (PDB: 1VBH) is shown with its nucleotide binding domain, linker region, and PEP binding domain coloured in cyan, pink, and brown, respectively. The PPDK Mg^2+^ is shown as a green sphere and its bound PEP as yellow sticks. (B) Section of the primary sequences in the structural alignment indicating the HMG/CHA aldolases essential Arg-123 in a magenta box, the PH with an orange sphere, and the PPDK threonine observed as a site of phosphorylation in plants as a green sphere. (C) Overlay of the HMG/CHA aldolase and EI:PTS with comparison of key residues. The enzymes are coloured as in panel A except, for clarity, the conserved domain in EI:PTS is coloured blue.

DALI was utilized to search for proteins containing similar structural fold as the αββα fold the HMG/CHA aldolase [31]. The aldolase shares structural similarity to the PH domain of the PT proteins and a domain of unknown function in bacterial pyruvate kinase (PK) (Fig 3A) [32,33]. A threonine residue in plant PPDK proteins (Thr-456 in Maize PPDK) is known to be reversibly phosphorylated and acts to regulate of PPDK function [34]. The HMG/CHA aldolase Arg-123, which is an essential residue for aldolase activity acting to stabilize the pyruvate enolate intermediate, structurally aligns between the conserved PH and phospho-threonine in PT proteins (Fig 3B) [22,23]. All of these residues are found in a loop region following a β-strand (β5 in the HMG/CHA aldolase) with the loop leading to an α-helix (α5 in the HMG/CHA aldolase) (Fig 3C). From the β-strand, the loop leading to the PH in PT proteins wraps from the opposite direction to that of the aldolase and leads to the reversibly phosphorylatable threonine found in the PT proteins where its side chain projects into the PT protein’s PEP binding site. The PH in the PT proteins directly precedes the α-helix whereas the Arg-123 in the aldolase is separated from the α-helix by the metal binding residue Asp-124. The aldolase Arg-123 is parallel, with the guanidinium inline with the helical dipole from α4. Whereas the PH is on the opposite side of the helical dipole and in all structures of PT proteins the imidazole of PH projects almost 180° away from the aldolase pyruvate binding site and into the characterized PT PEP binding site. The aldolase Gly-101, which lines the pocket of the pyruvate binding site, is found as a threonine/serine (Thr-164 in EI:PTS from *E*. *coli*; Ser-432 in PPDK from *Clostridium symbiosum*) in the PT proteins. The larger side chain in the PT enzymes would sterically restrict the PH residue from projecting into an equivalent position as the Arg-123 in the aldolase pyruvate binding site. Similarly, an additional loop region in the HMG/CHA aldolase (Arg-138 to Pro-156) would restrict the movement of Arg-123 preventing the residue from rotating into an equivalent position to the PH seen in the PT proteins. Together, the analyses indicate that the phosphorylatable residues of PT proteins and the aldolase Arg-123 are distinct, and whose functionalities operate on separate faces of a common fold. None of the residues of HMG/CHA aldolase pyruvate binding pocket are conserved in the PT proteins and none of the residues of the PT phosphoryl transfer pocket are conserved in the HMG/CHA aldolase. Thus, although phosphate binding and the reaction chemistries in these proteins are mediated through residues that are close in sequence space, significant differences in the protein structures leads to differences in the positions of the bound P_*i*_ among the proteins.

## Discussion

P_*i*_ is found to activate the HMG/CHA aldolase reaction, increasing the *k*_cat_ of the *P*. *putida* F1 enzyme by ~10-fold for the aldol substrates. The rate enhancement for CHA is slightly lower than that of the *P*. *straminae* enzyme which was activated ~50-fold in the presence of P_*i*_. However, in both versions of the enzyme, P_*i*_ acts to increase both the *k*_cat_ and *K*_M_ to similar degrees yielding similar catalytic efficiencies which is indicative of uncompetitive activation [8]. The concomitant increase in kinetic parameters is observed with all substrates and with both the aldolase and OAA decarboxylase activities. Uncompetitive activation is not a common occurrence amongst enzymes and the way the activators mediate activation appears to be varied. For example, the mouse alkaline phosphatase and bovine rod photoreceptor-specific ABC transporter are uncompetitively activated by the molecules *N*-ethylaminoethanol and all-*trans*-retinal, respectively. They are thought to bind to allosteric sites that modulate the enzyme active site and reaction kinetics [35,36]. In contrast, myrosinase, an *S*-glycosidase that catalyzes the cleavage of glucosinolates, utilizes its activator, ascorbate, directly in the reaction mechanism. Ascorbate binds in the aglycon binding pocket and activates the rate limiting second half of the ping-pong mechanism [37,38].

The OAA decarboxylase catalytic reaction does not require a general base but maintains the pyruvate enolate intermediate that requires protonation by a general acid to complete the reaction cycle. The P_*i*_ activation of both the aldolase and OAA decarboxylase activities suggests that the polyatomic ion contributes to the enzyme mechanism through activating the general acid half reaction. The rate enhancement of the aldolase’s pyruvate methyl proton exchange rate is in accordance with this reasoning where P_*i*_ enhances the rate > 300-fold. The pyruvate methyl proton exchange rate is likely slower than that of the true general acid half reaction as the exchange rate requires that the methyl proton be exchanged with the bulk solvent and not returned to enolate, which likely occurs to some degree. Thus, the large exchange rate enhancement by P_*i*_ is likely a result of P_*i*_ acting directly as the general acid which donates a deuteron to the pyruvate enolate, enhancing a rate that is not fully observed by the pyruvate methyl proton exchange assays. In the absence of P_*i*_, the OAA decarboxylase turnover rate (2 s^-1^) is slower than that of the aldolase reactions (> 10 s^-1^). The smaller rate enhancement in the OAA turnover rate in the presence of P_*i*_ likely suggests that in the decarboxylase reaction that the protonation of the pyruvate enolate is not the rate determining step, but rather it is the generation of the pyruvate enolate through decarboxylation that is rate limiting. Thus, it is likely that in the aldolase reaction the general acid step is rate determining which changes in the presence of P_*i*_ to the general base half reaction.

Docking studies suggested a binding site (site 1) close to, or overlapping, the proposed general acid Wat4 binding site. The residues potentially involved in the proposed P_*i*_ binding site were also shown to be important for the functioning of the aldolase. Significantly, substitution of either Asn-71 or Lys-147 with alanine decreased the *k*_cat_ of the HMG catalyzed reaction by > 1000-fold indicating important roles for these residues in the enzyme’s catalytic mechanism. Asn-71 and Lys-147 have been proposed to be involved with binding of the C5-carboxylate on CHA [19], a moiety not present on the HMG substrate, and whose residue substitutions were not hypothesized to make significant effects on the kinetics of the HMG catalysis. Significant reductions in the P_*i*_ activation in the N71A, K147A and other enzyme variants of residues comprising site 1 supports the role for these residues in facilitating P_*i*_ binding leading to activation of the general acid.

Only P_*i*_ and its analogs AsO_4_^3-^ and VO_4_^3-^ could activate the aldolase. The lack of activation by neither SO_4_^2-^ nor MoO_4_^2-^ indicates that the ion must also be in a suitable protonation state to facilitate the activation. The *P*. *straminea* HMG/CHA aldolase optimum pH increased from 6.6 to 8.2 where the dianionic form of the P_*i*_ (p*K*_a_ of values of P_*i*_ being 2.1, 7.2, and 12.7) would be the predominant and activating species [8]. The P_*i*_ activation of the aldolase through the general acid half reaction could be envisioned through a “proton switch” mechanism where P_*i*_ acts directly as the general acid, donating a proton, and simultaneously abstracts a proton from a source in the active site pocket [39]. In the structure of the HMG/CHA aldolase, the proposed general acid Wat4 is 2.8 Å from the pyruvate methyl group and is in the plane of the pyruvate molecule [19]. The source of the proton for the general acid should be out of the plane of pyruvate and may not be contributed from Wat04 but rather from bulk solvent. Thus, should P_*i*_ act directly as the general acid then Wat4 may serve as a ready source of a proton for the potential proton switch mechanism.

None of the other class II pyruvate aldolases are activated by P_*i*_, or by any other molecules, and the P_*i*_ activation appears to be unique to the HMG/CHA aldolase. P_*i*_ has also not been reported to activate any of the other enzymes from either the gallate or protocatechuate 4,5-cleavage pathways. The intracellular concentration of P_*i*_ in the Pseudomonads and Sphingomonads which contain these pathways is likely to be in the low millimolar range and would contribute to the aldolases normal physiological functioning [40]. In the gallate degradation pathway of *Pseudomonas putida*, the turnover rate of the enzymes are comparable to that of the native HMG/CHA aldolase (GalA = 42 s^-1^, GalD = unknown, and GalB = 2 to 14 s^-1^ depending on the metal utilized) [41,42]. Changes in cellular P_*i*_ content would likely not affect gallate utilization. However, the turnover rate of the enzymes from the protocatechuate 4,5-cleavage pathway in *Sphingomonas paucimobilis* SYK through degradation of protocatechuate are significantly higher than that of the aldolase in the absence of P_*i*_ (LigAB = 216 s^-1^, LigC = 260 s^-1^, LigI = 342 s^-1^, and LigJ = 277 s^-1^) [43–46]. Changes in cellular P_*i*_ content may be significant in controlling the flux through the protocatechuate pathway by activation of the aldolase.

The PH domain in PT proteins is similar in overall structure to the HMG/CHA aldolase (RMSD ~ 3Å). Essential residues in both the HMG/CHA aldolase and PT proteins also map to similar positions in the protein fold making an enticing suggestion for a similar mode of P_*i*_ binding. However, close inspection of structures of the enzymes indicate that the PT and aldolase active sites are on opposite faces of the protein fold. Further, there is little sequence conservation amongst the aldolase and PT proteins in their respective phosphate binding pockets. Gene copies of the HMG/CHA aldolase are commonly found in species of bacteria and plants which lack homologous genes to protocatechuate and gallate gene clusters, and the functional role of these proteins in these species unknown [21]. Although the overall sequence identity amongst HMG/CHA aldolase homologs is low, the proteins contain significant sequence conservation in the aldolase active site and the homologs likely support a class II pyruvate aldolase function. Thus, the presence of an HMG/CHA aldolase maintained across these species likely suggests that the aldolase have not recently evolved and that the PT proteins and HMG/CHA aldolase likely diverged in function a significant time ago resulting in non-conserved ligand binding sites.

## Supporting Information

S1 Fig The increase in the HMG/CHA aldolase catalyzed pyruvate methyl proton exchange rate due to increasing P_*i*_. (PDF)

S1 Table Primer sequences utilized in site-directed mutagenesis. (PDF)
