# Supplementary material for: Investigation into the Mode of Phosphate Activation in the 4-Hydroxy-4-Methyl-2-Oxoglutarate/4-Carboxy-4-Hydroxy-2-Oxoadipate Aldolase from Pseudomonas putida F1
Source: PLoS One. 2016 Oct 14;11(10):e0164556. doi: 10.1371/journal.pone.0164556 (PMC5065237; doi:10.1371/journal.pone.0164556)
Supplement: S1 Fig — (PDF) [file pone.0164556.s001.pdf]

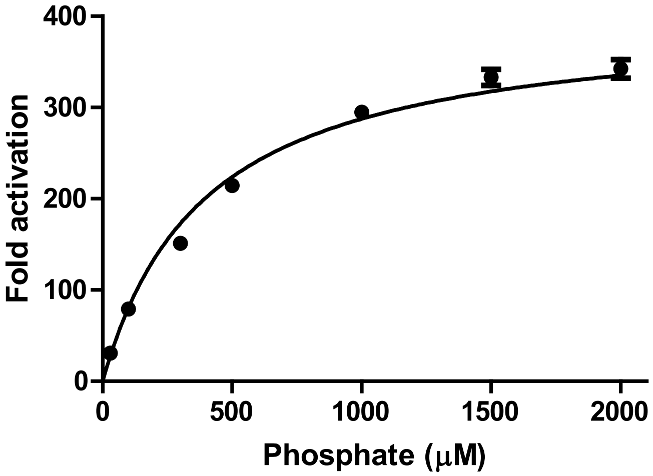

Fig S1. The increase in the HMG/CHA aldolase catalyzed pyruvate methyl proton exchange rate due to increasing P_i. The fold increase in activity plotted is relative to the rate in the absence of P_i ($2\ s^{-1}$).
