# Supplementary material for: Investigation into the Mode of Phosphate Activation in the 4-Hydroxy-4-Methyl-2-Oxoglutarate/4-Carboxy-4-Hydroxy-2-Oxoadipate Aldolase from Pseudomonas putida F1
Source: PLoS One. 2016 Oct 14;11(10):e0164556. doi: 10.1371/journal.pone.0164556 (PMC5065237; doi:10.1371/journal.pone.0164556)
Supplement: S1 Table — (PDF) [file pone.0164556.s002.pdf]

Table S1. Primer sequences utilized in site-directed mutagenesis.

Aldolase variant	Primer Sequences
R40A	CAAAACGCGAAAGGTCTGTTGTC
	CCTTTCGCGTTTTGAGCCTCATG
N71A	GGCGACGCTTGGATGTTTCATGTAGCCGTAGAGC
	CATCCAAGCGTCGCCAGGAGCTACGAGAAC
H75A	CGGCTACAGCAAACATCCAATTGTCGCCAGGAGCTACGAGAACAG
	GGATGTTTGCTGTAGCCGTAGAGCAGTGTCGG
R123K	GGGTGTCCTTGACTCCTGCATCAACAATCAGGGCTCGTACTC
	GGAGTCAAGGACACCCAGACGTTACGCGACATGGGGTTC
G144V	CACAAGTCACTGTCAAGGAAACACTC
	CAGTGACTTGTGCATTGATGGCTC
T145A	CAAGGCGCTGTCAAGGAAACACTCGGCTC
	CTTGACAGCGCCTTGTGCATTGATGGCTC
K147A	CTGTCGCGGAAACACTCGGCTC
	GTGTTTCCGCGACAGTGCCTTGTG
R195A	GGCCAGCGGCTTCGGCTGCAGCTACTAGAGTGC
	GCAGCCGAAGCCGCTGGCCTAGAAGAGGAAAAGCG
